# An Integrated Epigenomic and Genomic View on Phyllodes and Phyllodes-like Breast Tumors

**DOI:** 10.3390/cancers14030667

**Published:** 2022-01-28

**Authors:** Juergen Hench, Tatjana Vlajnic, Savas Deniz Soysal, Ellen C. Obermann, Stephan Frank, Simone Muenst

**Affiliations:** 1 Institute of Medical Genetics and Pathology, University Hospital Basel, 4031 Basel, Switzerland; juergen.hench@usb.ch (J.H.); tatjana.vlajnic@usb.ch (T.V.); savas.soysal@clarunis.ch (S.F.); 2 Visceral Surgery Research Laboratory, Clarunis, Department of Biomedicine, University of Basel, 4031 Basel, Switzerland; stephan.frank@usb.ch; 3 Department of Surgery, Clarunis University Center for Gastrointestinal and Liver Diseases Basel, 4031 Basel, Switzerland; 4 Institute of Pathology, Cantonal Hospital Lucerne, 6000 Lucerne, Switzerland; ellen.obermann@luks.ch

**Keywords:** fibroepithelial breast lesions, phyllodes tumors, methylation analysis, copy number alterations, dimension reduction, unsupervised machine learning

## Abstract

**Simple Summary:**

Fibroepithelial tumors of the breast represent a spectrum of mostly benign diseases. However, some of these tumors tend to recur and may even spread distantly to other body sites. Prediction of their biological behavior is currently morphology-centered. In this study, we set out to answer the question of whether their biologic behavior might be reflected by specific DNA methylation and copy number profiles, both of which can be determined alongside each other in a diagnostic routine workflow through microarrays. We discovered that the fibroepithelial tumors seem to fall into two distinct copy number variant patterns and that they are epigenetically related. Our study underlines the diagnostic usefulness of combined methylation/copy number profiling in fibroepithelial breast tumors to predict clinical outcomes.

**Abstract:**

Fibroepithelial lesions (FL) of the breast, in particular, phyllodes tumors (PT) and fibroadenomas, pose a significant diagnostic challenge. There are no generally accepted criteria that distinguish benign, borderline, malignant PT and fibroadenomas. Combined genome-wide DNA methylation and copy number variant (CNV) profiling is an emerging strategy to classify tumors. We compiled a series of patient-derived archival biopsy specimens reflecting the FL spectrum and histological mimickers including clinical follow-up data. DNA methylation and CNVs were determined by well-established microarrays. Comparison of the patterns with a pan-cancer dataset assembled from public resources including “The Cancer Genome Atlas” (TCGA) and “Gene Expression Omnibus” (GEO) suggests that FLs form a methylation class distinct from both control breast tissue as well as common breast cancers. Complex CNVs were enriched in clinically aggressive FLs. Subsequent fluorescence in situ hybridization (FISH) analysis detected respective aberrations in the neoplastic mesenchymal component of FLs only, confirming that the epithelial component is non-neoplastic. Of note, our approach could lead to the elimination of the diagnostically problematic category of borderline PT and allow for optimized prognostic patient stratification. Furthermore, the identified recurrent genomic aberrations such as 1q gains (including MDM4), CDKN2a/b deletions, and EGFR amplifications may inform therapeutic decision-making.

## 1. Introduction

Phyllodes tumor (PT), a rare breast neoplasm, accounts for 0.3% to 1% of all breast tumors [1]. The World Health Organization (WHO) classification (2019) currently divides PT into categories of benign (up to 75% of all PT), borderline, and malignant, based on a combination of several histologic features such as stromal cellularity, nuclear atypia, mitotic activity, stromal overgrowth, and delimitation of the tumor [1,2]. However, this morphology-based classification remains challenging, as there is considerable overlap between categories. Furthermore, diagnostic criteria cannot always be sufficiently appreciated on small biopsies. Given the resulting interobserver variability, the diagnosis of PT, and, in particular, the distinction between benign PT and fibroadenoma (FA) as well as between benign and borderline PT remains problematic in diagnostic routine [1]. Furthermore, the differentiation of malignant PT from metaplastic carcinoma or primary breast sarcoma is not straightforward either [3]. Studies have shown that the overall rate of concordantly diagnosed FA and benign PT lies between 40 and 60% [1]. Additionally, histological grading correlates with prognosis but is not predictive of clinical behavior in all patients [1]. At the moment, no clinically applicable biomarkers exist, and the pathogenesis, as well as the molecular background of PT, remain largely unknown [1].

While benign PT have a low recurrence risk (10–17%), borderline and malignant PT tend to relapse in a significant proportion of patients (14–25% and 23–30%, respectively), justifying surgical excision with tumor-free margins of 10 mm [3]. Moreover, malignant PT metastasize in up to 29% [4], most commonly to the lungs and skeleton, invariably indicating a dismal prognosis [3]. Molecular characteristics that conclusively distinguish between FA, benign, borderline, and malignant PT, as well as breast carcinomas and primary breast sarcomas, would, therefore, satisfy an urgent, currently unmet clinical need in breast surgery.

In recent years, combined genome-wide DNA methylation and chromosomal copy number analysis by microarrays has gained considerable interest as a precise tool to classify benign and malignant tumors based on their individual, often lineage-reflecting methylation patterns [5,6,7,8]. Most prominently, the brain tumor methylation classifier has become a mainstay in neuropathological tumor diagnostics worldwide [5] and has already influenced several entity definitions in the 2016 WHO classification [9]. It has recently also been adopted for soft tissue tumors [10] and outperforms histology not only in precision but also diagnostic speed when applied to intraoperative cryo specimens, employing nanopore sequencers instead of microarrays [11,12]. The wealth of methylation data in public repositories allows unsupervised machine learning [13,14] approaches to cross-compare a single diagnostic case against thousands of other specimens [15,16,17]. As opposed to supervised machine learning-based static classifiers [5,7,10,18,19,20], unsupervised approaches are able to place data series extraordinarily rare tumors [21] in the context of a magnitude of neoplastic and non-neoplastic differentiation based on the raw data alone [12,17]. In addition to fine-tuned supervised machine learning [22], integrated interpretation of copy number alterations, genetic changes, and histology can significantly increase disease course prediction granularity [23,24].

Given their morphological and immunohistochemical characteristics, we hypothesized that PT and FA represent a fibroepithelial lesion (FEL) spectrum originating from similar or identical cells of origin affected by different initial genomic damage events. Similar observations have been made, e.g., in meningiomas [20,25] and primary brain tumors [5,26]. Methylome detection tools, in particular, microarrays (Infinium bead chip arrays, Illumina, San Diego, CA, USA) deliver both methylation signatures and genome-wide copy number profiles [6,11], providing a dual use for routine diagnostics. To test our hypothesis and simultaneously generate backbone reference data to train machine learning systems, we employed the more comprehensive methylation array strategy.

## 2. Materials and Methods

### 2.1. Tissue Collection

After identifying potential samples from FA, PT, breast carcinomas (BC), and primary breast sarcomas (BS) in the biobank at the Institute of Medical Genetics and Pathology, University Hospital Basel, an H&E-stained cryo-section of the freshly frozen (FF) tissue was prepared, and diagnosis in each case was re-confirmed by a specialized breast pathologist (S.M.) by reviewing the frozen section, or, where available, FFPE slides. FF tissue of 37 samples (1 FA, 30 PT, and 6 BS), diagnosed between 1990 and 2017, was included. Furthermore, FFPE specimens from 23 cases (2018–2020) from the archives of the Institute of Medical Genetics and Pathology, University Hospital Basel and the Institute of Pathology, Cantonal Hospital, Lucerne (10 FA, 12 PT, and 1 metaplastic BC) were analyzed. The respective H&E-stained sections were also reviewed and the diagnosis was confirmed by an expert breast pathologist (S.M.). This study was approved by the Ethikkommission Nordwest- und Zentralschweiz (EKNZ, proposal number 2014-397 and PB_2020-00071). The study was performed in accordance with the Declaration of Helsinki.

### 2.2. Methylation and Copy Number Analysis

The technology is based on a beadchip microarray (Infinium human methylation EPIC, by Illumina, San Diego, CA, USA), consisting of a modified single nucleotide polymorphism (SNP) array to quantify DNA methylation. The current array covers approx. 850,000 CpG islands distributed across the entire genome. The procedure is well-established and part of our routine diagnostic practice: of each case, depending on biopsy size and tissue cellularity, 2 to 6 cryosections (70 µm) or 7 to 15 FFPE sections (4 µm) were used for DNA isolation (Maxwell FFPE kit, Promega, Madison, WI, USA). DNA was quantified by absorption measurement (NanoDrop, Thermo Fisher, Waltham, MA, USA). After bisulfite-conversion and low-level amplification, the DNA was hybridized to beadchips which are then read on an appropriate scanner (typically iScan, service provided by Life&Brain, Bonn, Germany). The resulting data (IDAT format) were then preprocessed and normalized (SWAN), mapped to the genome, and converted into beta values (which represent methylation state at each scanned site; all preprocessing via minfi) [27,28]. Top differentially methylated probes were determined by calculation of standard deviations across the entire dataset comprising >18,000 cases obtained from public resources including TCGA and Gene Expression Omnibus (GEO), as well as from in-house reference collections. The 75,000 probes with the highest standard deviations were selected (File S2). This filtered set of methylation beta values was then compared by uniform manifold approximation projection (UMAP) for dimension reduction as previously described [13,15]. This resulted in an unsupervised, bias-free grouping of samples sharing similar DNA methylation patterns, which often reflect individual (biological) entities [5]. Of note, probes were not selected based on their annotation to specific genes. Copy number plots were generated with the conumee [29] in R. R 3.6.3 on Ubuntu Linux 18.04 (x86_64) was used throughout this study.

### 2.3. Fluorescent in Situ Hybridization (FISH)

After deparaffinization and hydration of 3–4 µm-thick slides, sections were further processed for FISH according to our in-house protocol. In brief, the slides were pretreated automatically with the Leica Bond-III (Leica Microsystems, Wetzlar, Germany), then manually washed with water and dehydrated by 70%, 80%, and 100% ethanol. Subsequently, slides were incubated overnight with commercially available SPEC CDKN2A/CEN 9 and SPEC RB1/13q12 Dual Color Probe kits (ZytoVision, Bremerhaven, Germany) as well as LSI EGFR SpectrumOrange/CEP7 SpectrumGreen (Abbott, Chicago, IL, USA) probes.

### 2.4. Nanopore Sequencing

Tumor DNA was sequenced with the RAD SQK-004 sequencing kit on a FLO-MIN106D (R9.4.1) flow cell mounted on a MinION Mk1B sequencing device (Oxford Nanopore Technologies, Oxford, UK). Sequencing was controlled by the MinKNOW (core: 4.1.2, guppy: 4.2.2, bream: 6.1.4, script conf. 4.1.15; platform ARMv8; distribution: MinIT; Oxford Nanopore Technologies, Oxford, UK) through NanoDiP [12] which controls MinKNOW through the MinKNOW API. Sequencing and data processing were carried out on a Jetson AGX Xavier 32GB developer kit (NVIDIA, Santa Clara, CA, USA).

## 3. Results

### 3.1. Patient Characteristics

In total, the tissue of 41 PT was available for analysis. All patients with PT were female, and the mean age at diagnosis was 51.7 years (a range from 14 to 86 years). All tumors were located in the breast, with the exception of one specimen, which was from a cerebellar metastasis of a malignant PT. Of the 41 analyzed tumors, 22 had an initial histologic diagnosis as benign, 8 as borderline, and 10 as malignant PT, respectively. In one case from 1990, the diagnosis was “phyllodes tumor” without further specification. The initial diagnosis was confirmed in all cases by an experienced breast pathologist (S.M.).

### 3.2. Patient Outcome

Follow-up was available for 19 patients (mean follow-up time 75.8 months, range 6–219 months). Of the nine patients with the diagnosis of a benign PT, six were disease-free postoperatively, and three had recurrent disease: one patient after 45 months, with a second recurrence 19 months later, and the other two after 34 and 96 months, respectively. All three recurrent PT showed the same histology as the primary tumor. All 5 patients with a borderline PT remained disease-free. Of the five patients with a malignant PT, three showed no evidence of disease, one patient presented with recurrent disease after 6 months, and one patient had a cerebellar metastasis after 36 months, with no further follow-up available after this event.

### 3.3. DNA Methylation and Copy Number Changes

**Figure 1:**
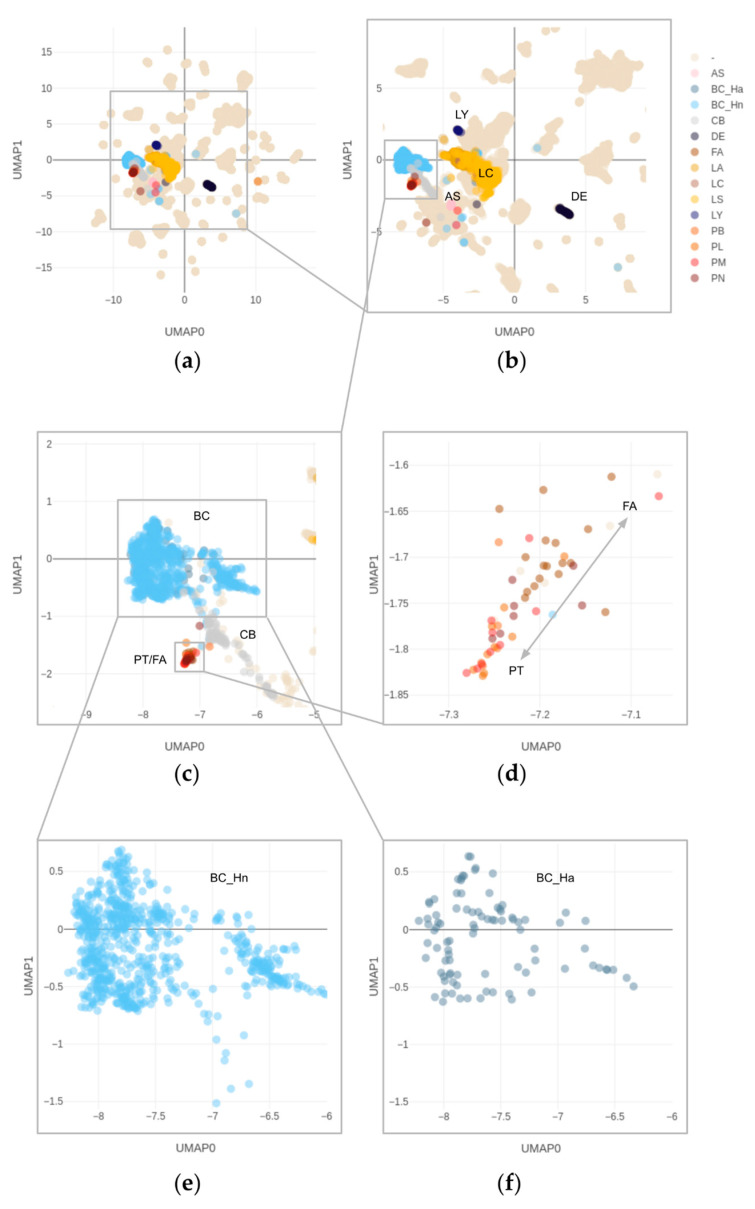
UMAP plot of the combined TCGA, GEO, and in-house data reference set alongside phyllodes tumor specimens, zoomed-in view. The specimens are annotated as follows: AS = angiosarcoma; BC_Ha = breast adenocarcinoma, Her-2 amplified; BC_Hn = breast adenocarcinoma, Her-2 not amplified; CB = control breast; DE = degraded DNA; FA = fibroadenoma; LA = lung adenocarcinoma; LC = lung cancer, NOS; LS = lung squamous cell carcinoma; LY = diffuse large B cell lymphoma; PB = PT borderline; PL = PT benign; PM = PT malignant; PN = PT, NOS. Note that phyllodes tumors cluster in the vicinity to reference control breast tissue (CB) but form a distinct methylation class. The non-annotated cases in this plot, designated “-” represent all remaining specimens registered in the EpiDiP.org platform, mostly comprising TCGA and GEO datasets. They have been omitted from zoomed plots for clarity. An interactive (zoomable, annotated) plot can be found in File S1. In addition, the plot coordinates are provided with Sentrix ID annotation in XLSX (MS Excel) and RDA (R 3.6.3) format. In addition, all cases, including their copy number, profiles may be viewed on www.epdip.org (accessed on 13 January 2022), see instructions on our platform’s website. (**a**) overview, (**b**–**f**) magnified subsets from (**a**), unannotated cases hidden (**c**–**f**).

We included a total of 18,537 methylome profiles, the majority of which were available through TCGA and GEO (Figure 1, Files S1, S3, S4). According to the annotation, these comprise 854 “breast cancer” (BC) and 97 “control breast” (CB) samples. While the majority of BC samples showed high-amplitude CNVs (769/854; 90%) and mostly clustered together with BCs from our cohort (Figure 1), a few cases (12/854; 1%) clustered with CB, likely representing BC samples with low tumor cell content as reflected by their low CNV amplitudes. No CB-annotated cases clustered with the high-amplitude CNV BCs. Out of high-amplitude BCs, a minority (94/769; 12%) showed an ERBB2 gene amplification; these cases did not form a separate cluster within BCs (Figure 1, Files S1, S3, S4).

In addition, 2 primary breast angiosarcomas and 1 metaplastic breast carcinoma were included to test whether they would fall into the respective reference data clusters. Indeed, the angiosarcomas as well as the metaplastic carcinoma clustered in their respective groups, and no overlap with the PT was found (Figure 1, Files S1, S3, S4).

Interestingly, a fresh frozen sample of a 79-year-old patient, initially diagnosed as a malignant PT, showed a methylation profile consistent with diffuse large B-cell lymphoma (DLBCL). This case dated back to the pre-immunohistochemistry era, and retrospective immunohistochemical work-up of available FFPE tissue indeed confirmed the diagnosis of DLBCL (CD20 positive and CD5 negative).

### 3.4. Overlapping Methylation Patterns of Phyllodes Tumors and Fibroadenomas

Interpretable methylation array data could be obtained for 38 of the 41 analyzed PT specimens while the remaining three samples clustered as “degraded DNA” array samples.

Moreover, 51 tumors, comprising 34 PT and all 17 FA, formed a methylation cluster in proximity to the BC and CB clusters, and distinct from adenocarcinomas of the breast (Figure 1). Likewise, the PT/FA methylation pattern differed from non-neoplastic breast tissue but was more similar to the latter. This is reassuring since the majority of neoplasms that do not carry driver alterations within strong epigenomic modifiers (e.g., IDH1/2, SMARCB1) largely retain epigenomic features of their precursor lineages [17]. The remaining four tumors histologically diagnosed as PT clustered elsewhere in proximity to mostly mesenchymal tumors (Figure S1). The PT/FA cluster showed a slight trend to separate FA from PT, but overlap currently remains high and without a clear distinction between PT histologically diagnosed as benign, borderline, or malignant (Figure 1D).

### 3.5. Copy Number Alterations in Phyllodes Tumors and Fibroadenomas

**Figure 2:**
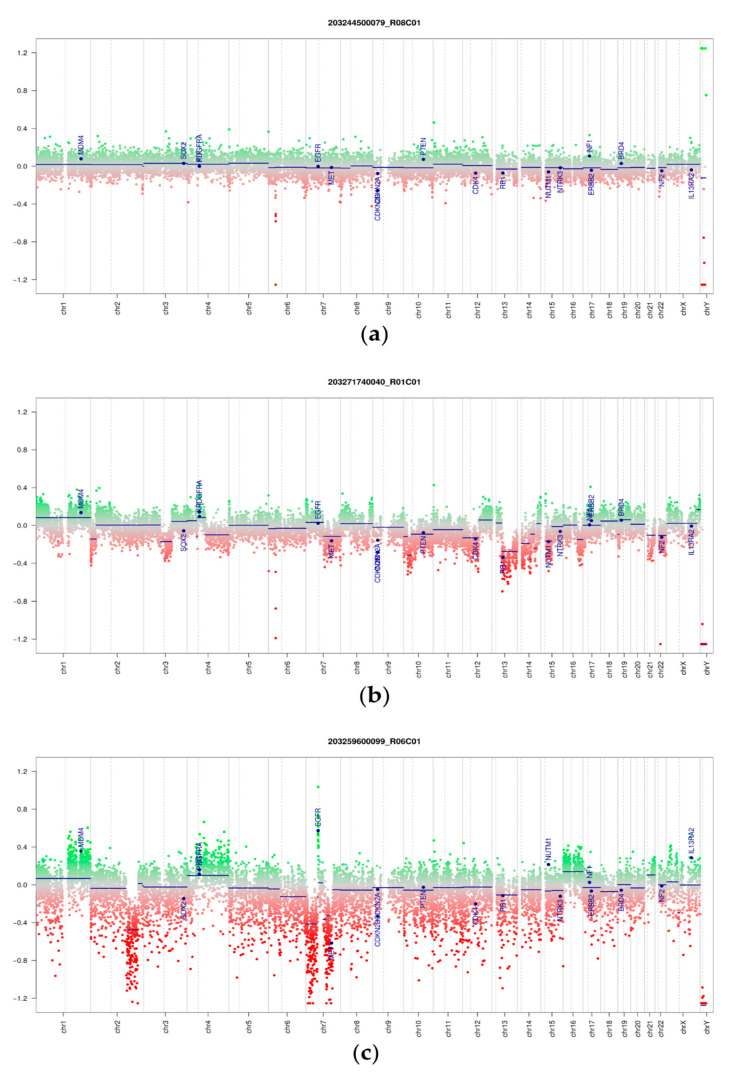
Genome-wide copy number variation profiles of 3 phyllodes tumors. (**a**) Benign phyllodes tumor. (**b**) Malignant phyllodes tumor. (**c**) Some phyllodes tumors, here a malignant form, show a potentially targetable EGFR gene amplification. The remaining copy number profiles of PT and FA can be accessed online [17] by searching for the respective Sentrix IDs. Gray full lines indicate chromosomal borders, dashed lines represent centromeres. Summary plots for CNV profiles can be found in File S6.

Copy number plots were computed from microarray data (representative examples in Figure 2). We visually classified copy number aberrations in a tumor type agnostic manner into four categories: flat, high-amplitude of CNV, low-amplitude of CNV, and degraded/unclear. In addition, we computed copy number summary plots from those cases with low high signal to noise ratios as previously described [5,30] (Figure S6. for plots, Figure S7 for list of all analyzed array files). The summary plots demonstrate a strong correlation between increased copy number alterations and morphological changes associated with malignancy. Table 1 summarizes the identified CNVs and available clinical data.

For the histologically classified FAs and benign PT, most tumors showed low-amplitude CNVs or flat copy number profiles (Table 1, Files S6, S7), with the exception of three cases (two benign PT and one FA) featuring high-amplitude CNV profiles with either CDKN2a/b deletion (two benign PT) or MDM4 gain (1 FA). Importantly, the histology of these three cases was concordant with the initial diagnosis. Of note, one histologically benign PT with CDKN2a/b deletion showed an increased proliferation rate as well as a strong expression of p53, both of which have been linked to malignancy in PT [31]; this patient was initially resected with a very close resection margin (<1 mm), and developed recurrent disease after 46 months, and then again after 19 months. The recurrent PT were both again excised with clear margins and featured benign histology in both instances. Further follow-up of the patient is not available. For the second benign PT with CDKN2a/b deletion, no recurrence has been recorded up to now. The FA with MDM4 gain was histologically unremarkable, and no recurrent disease was recorded. Interestingly, of the 4 PT histologically diagnosed as borderline, two showed high- and the other two low-amplitude CNV profiles, indicating that borderline PT do not seem to represent a distinct biological entity, but instead may be classified as either benign PT with a flat copy number profile, or malignant PT with high-amplitude CNVs.

CNV profile analysis also revealed recurrent genomic aberrations. In total, 17 PT showed a gain of 1q (including MDM4), while 6 PT featured a CDKN2a/b deletion, of which 4 were initially diagnosed as malignant (see above). Notably, of 9 PT with 1q gain and available follow-up, only one recurred after 6 months, while both PT with CDKN2a/b deletion and follow-up developed recurrent disease, in one case recurring twice over a period of 65 months.

**Figure 3:**
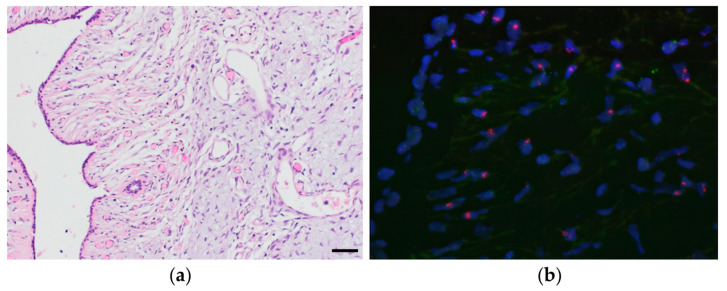
(**a**) H&E of a borderline PT (magnification 200×) and (**b**) corresponding FISH image (magnification 400×), which shows an EGFR gene amplification (red) in the stromal cells, but not the adjacent benign epithelium (top right). Green is the centromere probe for chromosome 7. Case ID: GSM5418510.

One of the malignant PT showed an RB1 deletion, and one patient with 2 unilateral PT (one classified as malignant, and one as PT not otherwise specified (NOS)/borderline in histology) harbored a potentially targetable epidermal growth factor receptor (EGFR) gene amplification in both tumors (Figure 3). In this patient, the malignant PT additionally showed Rb1 deletion, and the borderline PT showed an additional MDM4 amplification on top of the EGFR amplification. This suggests a joint tumor origin with divergent genetic aberrations, which in one case led to a malignant PT.

FISH analysis of PT cases with genomic aberrations was able to verify the CDKN2a deletions in three tumors, as well as the RB1 deletion in two tumors and the EGFR amplification in two tumors. In the remaining three cases with CDKN2a deletion, probe hybridization failed, probably due to the advanced age of the tissue. Importantly, all examined deletions and amplifications were only present in the stroma, and not in the adjacent epithelial cells, as illustrated in Figure 3.

### 3.6. Proof-of-Concept Experiment Using Nanopore Sequencing

Nanopore same-day diagnostics requires native tumor DNA [6,11] which was available for some archival specimens. To demonstrate the technical validity of nanopore sequencing as an ultra-fast alternative to microarrays, we ran an aliquot of a histologically malignant PT (GSM5418497) on NanoDiP [12]. Previously described run parameters [6,11] applied in daily brain tumor routine were applied without modification. A total of 150 megabases of high-quality reads were obtained in 2 h 5 min. Run analysis and details are included in the File S5. The resulting UMAP [13] plot based on 4488 CpG sites identified in 142,994 reads shows a methylation pattern equivalent to the one generated from array data (Figure 1, case identifier in Table 1), placing FLs close to breast control tissue samples, which in turn are located in proximity to invasive breast cancers. The copy number plot from nanopore read alignment recapitulates the aberrations determined in the microarray analysis File S5, compare Nanopore data B1992_24268_20211126_BC12_AllIDATv2_20210804_NanoDiP_report.pdf with microarray-based plot 203293640041_R07C01_CNV_IFPBasel_annotations.pdf).

## 4. Discussion

Our combined methylation and copy number analysis revealed that PT do indeed represent a biologically distinct group of breast neoplasms, and are part of a spectrum between normal breast tissue and invasive breast cancer; within the PT group, tumors form a gradient from benign (closely resembling benign breast tissue) to malignant. While the methylation profiles of PT and FA converge in a cluster distinct from BC and normal breast tissue, their copy number profiles prompt for a separation of the FA/PT tumor class into malignant (high CNVs) and benign (flat copy number profiles or few CNVs) forms. This suggests that tumors histologically categorized as “borderline” may not represent a distinct biological entity, but instead separate into benign and malignant PT as revealed by combined methylation and copy number analysis. Our molecular approach could thus be used to discriminate benign from malignant PT, especially in the diagnostically difficult borderline category, thereby aiding clinical patient management. Omitting the twilight category of “borderline” PT is likely to not only streamline the diagnostic process but may also contribute to an optimized diagnostic and prognostic patient stratification. This may help clinicians as well as patients to more confidently plan for potential revision surgery and follow-up, since borderline tumors with a flat CNV profile most likely follow a benign clinical course requiring no additional treatment, whereas borderline tumors with multiple copy number aberrations are potentially malignant, requiring wide excision as well as a close clinical follow-up.

Of note, confirmation of our result on the NanoDiP platform confirms the feasibility of our approach and underlines the straightforward clinical applicability of our FL reference data collection.

Our analysis also revealed recurrent CNV aberrations such as 1q gains, CDKN2a/b deletions, and MDM4 gains (Figure 2). Of note, these CNV aberrations were verified by FISH. Using a single microarray-based technique, or, alternatively, nanopore-based sequencing [11], both copy number and methylation profiles are obtained and evaluated simultaneously.

Interestingly, out of all PT with 1q gain and available follow-up (*n* = 9), only one recurred, while both PT with CDKN2a/b deletions developed recurrent disease. Truncating as well as non-synonymous CDKN2a mutations, as well as homozygous CDKN2a deletions, are known to occur in recurrent PT with histologically benign, borderline, and malignant characteristics [32,33]. These findings suggest that loss of CDKN2a gene function might underlie (or contribute to) PT recurrence, independent of histological grade, and indicate that CDKN2a analysis could be useful to identify patients at risk for recurrent disease.

One patient had two distinct PT at presentation (one borderline and one malignant histologically), both of which showed EGFR amplification, suggesting that these represent two clonally related but histologically distinct tumors. Detection of EGFR amplification by FISH has been described in up to 16% of PT and has been associated with tumor progression [34]. Unfortunately, no clinical follow-up is available for our patient.

Finally, both EGFR amplifications and CDKN2a/b deletions represent potentially targetable gene aberrations. Although EGFR amplifications were found in only two out of 41 PT samples, this alteration could represent a potential therapeutic target. With new anti-EGFR therapies and CDK4/6 inhibitors entering clinical practice, identification of these alterations may become part of the routine molecular diagnostic workup of PT tumors.

## 5. Conclusions

The distinct methylation and CNV signatures of the histological PT/FA spectrum not only allow the diagnostic discrimination of PT from histological mimics such as sarcomas or carcinomas, but also enable the distinction between benign and malignant PT. These findings may potentially eliminate the need for a borderline category, paving the way to optimized prognostic patient stratification and clinical management. Verification of copy number aberrations through FISH confirms that the stroma (as opposed to epithelial cells) represents the neoplastic component in PT. As recurrent genomic aberrations such as EGFR amplification and CDKN2a/b deletion may represent therapeutic targets, their diagnostic identification could impact the clinical management of recurrent or metastatic PT patients.

Lastly, while having analyzed only a single case so far with the diagnostic same-day nanopore sequencing [12] that our institution routinely applies in brain tumor diagnostics, we demonstrate the immediate clinical applicability of our FL reference data collection, which we are making publicly available alongside this manuscript.

## Figures and Tables

**Table 1:** Fibroepithelial lesions with clinical parameters, annotation, and copy number changes.

GEO ID	Sentrix ID	Histology	Methylation Category	Age at Diagnosis	Follow-Up (Months)	Recurrent Disease	CNV Aberrations	Confirmed by FISH
GSM5418497	203293640041_R07C01	malignant PT	PHYT_MAL	50	6	yes	1 gain	
GSM5418498	203271740040_R08C01	PT NOS	PHYT_NOS	64		NA	1q gain	
GSM5418499	203259060045_R04C01	benign PT	PHYT_NOS	59	81	no	1q gain	
GSM5418500	203259600099_R07C01	borderline PT	PHYT_BOR	65	30	no	1q gain	
GSM5418501	203259600099_R06C01	malignant PT	PHYT_MAL	72	91	no	1q gain	
GSM5418502	203271740040_R01C01	malignant PT	PHYT_MAL	21		NA	1q gain	
GSM5418503	203257020148_R07C01	malignant PT	PHYT_NOS	66	48	no	1q gain	
GSM5418504	203253040182_R07C01	malignant PT	PHYT_MAL	50	135	no	1q gain	
GSM5418505	203293640041_R06C01	malignant PT	PHYT_MAL	50	122	no	1q gain	
GSM5418506	203244490194_R06C01	borderline PT	PHYT_NOS	83	60	no	1q gain	
GSM5418507	203946830053_R07C01	benign PT	PHYT_NOS	40	15	no	1q gain	
GSM5418508	203836210043_R03C01	benign PT	PHYT_BEN	54			1q gain	
GSM5418509	203836210043_R04C01	malignant PT	PHYT_MAL	51		NA	1q gain, EGFR amp., RB1 del.	RB1 del., EGFR ampl.
GSM5418510	203836210043_R07C01	benign or borderline PT	PHYT_NOS	51		NA	1q gain, MDM4 amplification, EGFR amp.	EGFR ampl.
GSM5418511	203259600099_R05C01	benign PT	PHYT_BEN	69		NA	1q/MDM4 gain	
GSM5418512	203259060045_R01C01	benign PT	PHYT_BEN	60		NA	CDKN2a/b deletion	
GSM5418513	203271740040_R07C01	benign PT	PHYT_MAL	50		NA	CDKN2a/b deletion	
GSM5418514	203808570131_R05C01	malignant PT	PHYT_MAL	48	36	yes	CDKN2a/b deletion	CDKN2a/b deletion
GSM5418515	203271740040_R02C01	benign PT	PHYT_BEN	42		NA	MDM4 gain	
GSM5418516	203271740040_R03C01	benign PT	PHYT_BEN	46		NA	MDM4 gain	
GSM5418517	203259060045_R02C01	borderline PT	PHYT_NOS	82		NA	MDM4 gain, CDKN2a/b deletion	
GSM5418518	203271740040_R06C01	benign PT	PHYT_BEN	36	65	yes	MDM4 gain, CDKN2a/b deletion	CDKN2a/b deletion
GSM5418519	203271740040_R05C01	FA	BR_FAD	38			MDM4 gain, malignant-looking CNV	
GSM5418520	203293640041_R03C01	benign PT	PHYT_MAL	64	204	no	RB1 deletion	RB1 deletion
GSM5418521	203253040182_R08C01	malignant PT	PHYT_MAL	83		NA	susp. 1q gain (bad DNA)	

Table legend: PHYT_MAL = malignant phyllodes tumor, PHYT_BOR = borderline phyllodes tumor, PHYT_BEN = benign phyllodes tumor, PHYT_NOS = phyllodes tumor, not otherwise specified. The full list of all analyzed samples can be found in File S8.

## Data Availability

The datasets generated and/or analyzed during the current study are available in the GEO repository, https://www.ncbi.nlm.nih.gov/geo/query/acc.cgi?acc=GSE179458 (accessed on 13 January 2022). Furthermore, all data can be viewed in a processed manner on http://www.epidip.org (accessed on 27 May 2021). Individual cases can be identified by searching for their Sentrix ID. A comprehensive list of all specimens analyzed by microarray in the process of this study is included as File S8.

## Supplementary Materials

The following supporting information can be downloaded at: https://www.mdpi.com/article/10.3390/cancers14030667/s1, File S1: PTplot.html. Interactive UMAP plot with annotation of the methylation classes mentioned in this manuscript. Requires an up-to-date web browser; tested with Chrome and Firefox. For reference on how to zoom, search, hide, etc. refer to the plotly user manual; File S2: PT20210527_UMAP6_all_bVals_top_75000_cgList.rds.rda. CpG list in Illumina annotation in R 3.6.3 RDA format, refer to Illumina’s documentation concerning the genomic positions of the probes; File S3: PT20210527_UMAP6_all_bVals_top_75000.xlsx. Coordinates of the UMAP plot in MS XLSX format, annotated with Sentrix IDs; File S4: PT20210527_UMAP6_all_bVals_top_75000.rda. Coordinates of the UMAP plot in R 3.6.3 RDA format, annotated with Sentrix IDs; File S5: NanoDiP_PT.zip. Report and nanopore run characteristics of the proof-of-concept experiment (PDF and HTML files with plots); File S6: CNsummaryPlots.pdf. Contains summary plots of copy number profiles based on microarray data; case selection according to signal to noise ratio to meet the requirements of the CNsummaryplots analysis tool. List of included array IDs in File S7; File S7: CNsummary_files.xlsx. List of array IDs and specimen types included in the copy number summary plots (S6); File S8: PT_Table_ GEO_Repository.xlsx. List of all cases/IDs included in the Gene Expression Omnibus repository (see data availability statement).
