# Supplementary material for: An Integrated Epigenomic and Genomic View on Phyllodes and Phyllodes-like Breast Tumors
Source: Cancers (Basel). 2022 Jan 28;14(3):667. doi: 10.3390/cancers14030667 (PMC8833410; doi:10.3390/cancers14030667)
Supplement: Supplementary file 1 [file cancers-14-00667-s001.zip › S6 CNsummaryPlots.pdf]

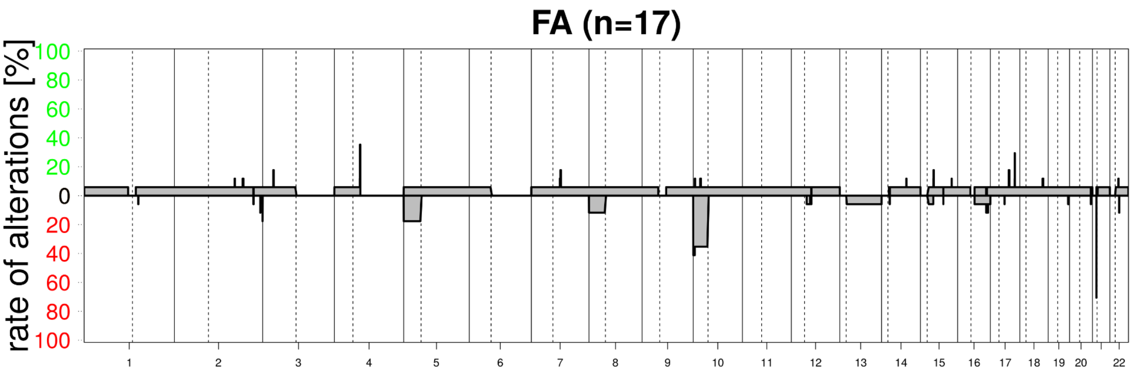
FA (n=17)
rate of alterations [%]
100 80 60 40 20 0 20 40 60 80 100
1 2 3 4 5 6 7 8 9 10 11 12 13 14 15 16 17 18 19 20 22

(a)

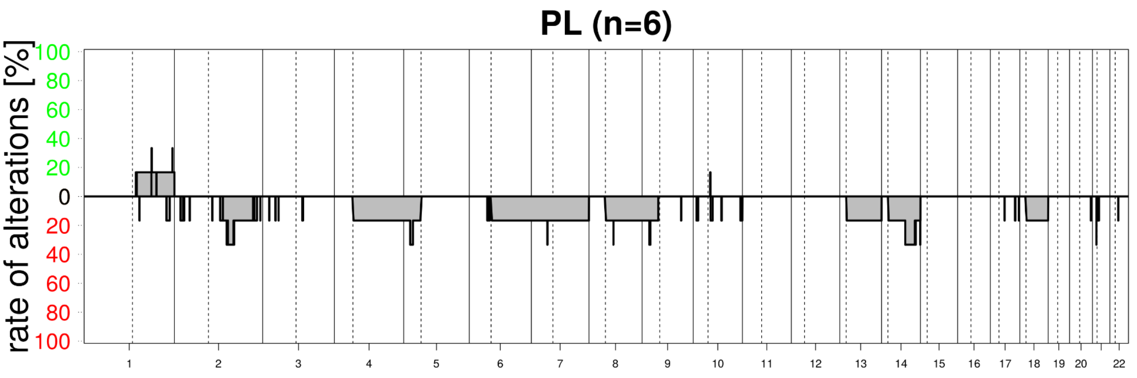
PL (n=6)
rate of alterations [%]
100 80 60 40 20 0 20 40 60 80 100
1 2 3 4 5 6 7 8 9 10 11 12 13 14 15 16 17 18 19 20 22

(b)

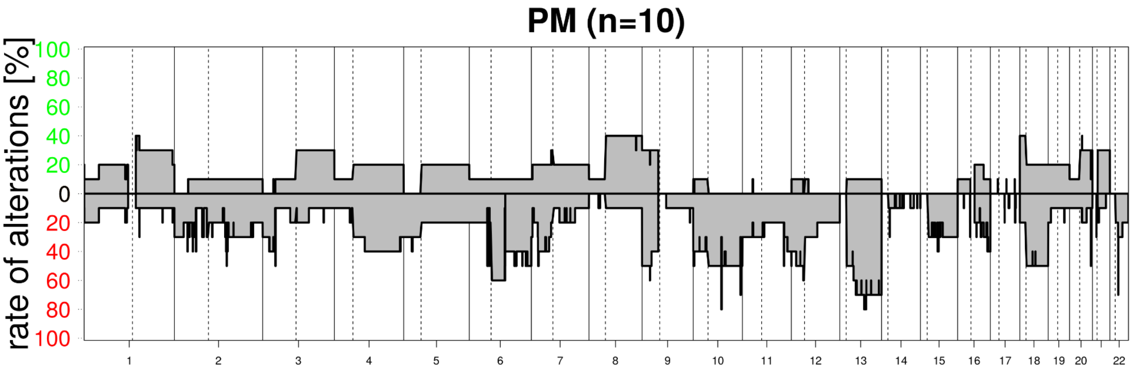
PM (n=10)
rate of alterations [%]
100 80 60 40 20 0 20 40 60 80 100
1 2 3 4 5 6 7 8 9 10 11 12 13 14 15 16 17 18 19 20 22

(c)

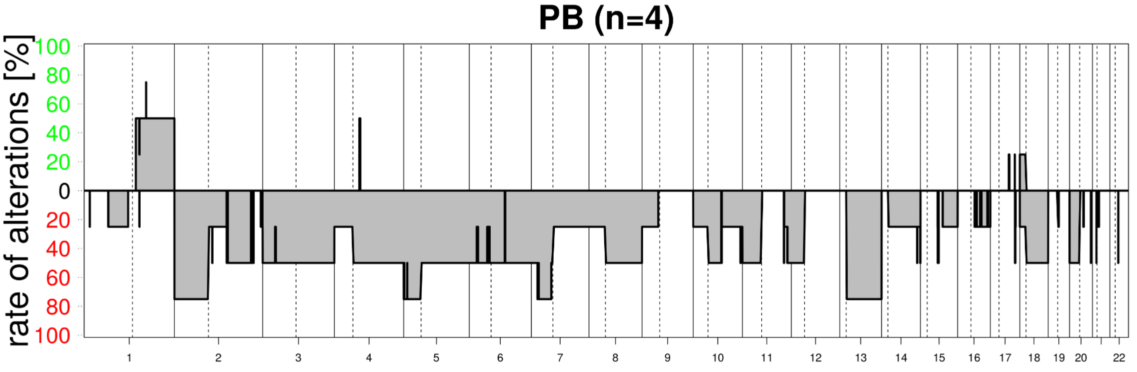
PB (n=4)
rate of alterations [%]
100
80
60
40
20
0
20
40
60
80
100
1
2
3
4
5
6
7
8
9
10
11
12
13
14
15
16
17
18
19
20
22

(d)

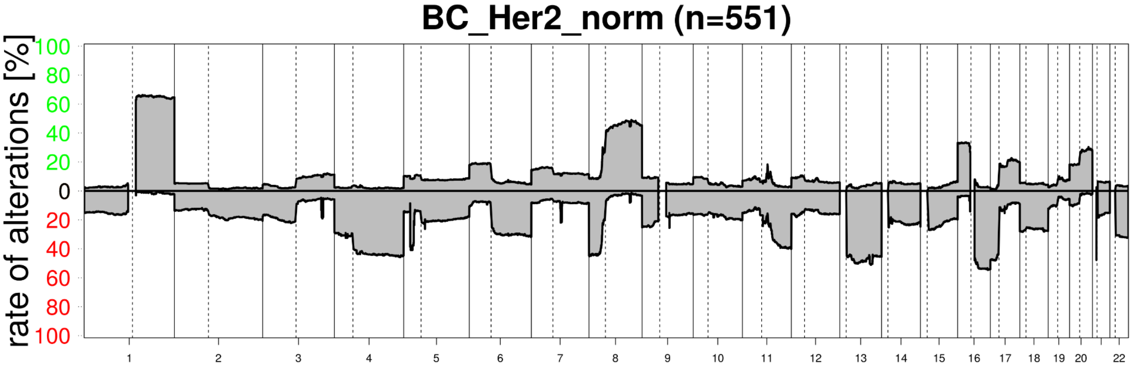
BC_Her2_norm (n=551)
rate of alterations [%]
100
80
60
40
20
0
20
40
60
80
100
1
2
3
4
5
6
7
8
9
10
11
12
13
14
15
16
17
18
19
20
22

(e)

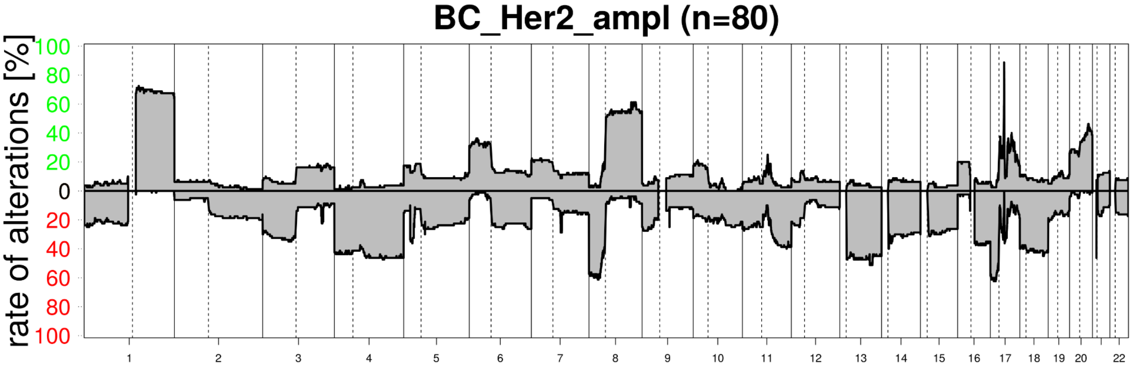
BC_Her2_ampl (n=80)
rate of alterations [%]
100
80
60
40
20
0
20
40
60
80
100
1
2
3
4
5
6
7
8
9
10
11
12
13
14
15
16
17
18
19
20
22

(f)

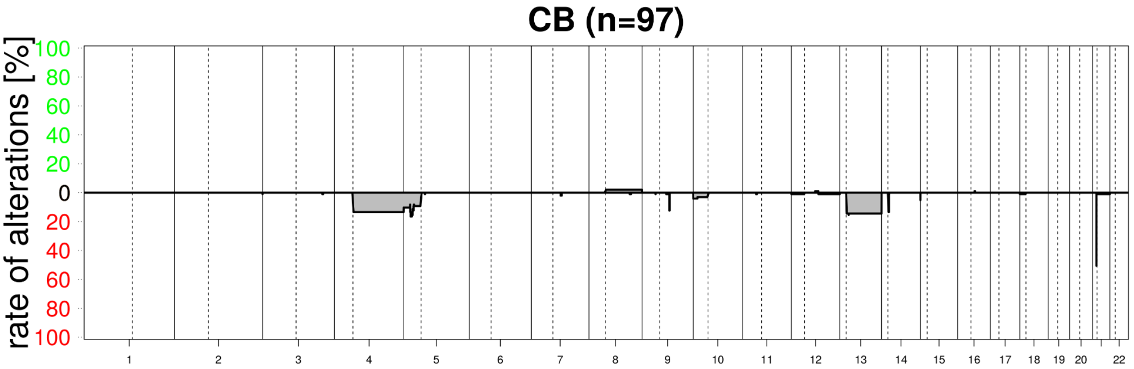
CB (n=97)
rate of alterations [%]
100
80
60
40
20
0
20
40
60
80
100
1
2
3
4
5
6
7
8
9
10
11
12
13
14
15
16
17
18
19
20
22

(g)

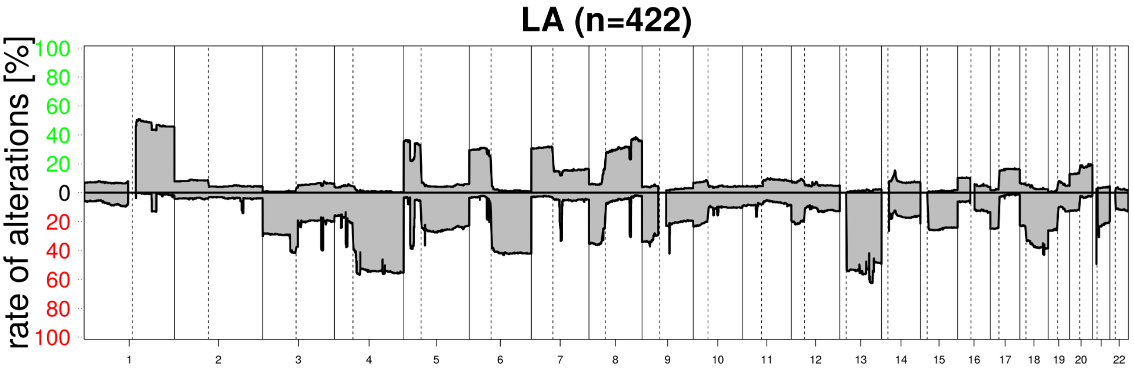
LA (n=422)
rate of alterations [%]
100
80
60
40
20
0
20
40
60
80
100
1
2
3
4
5
6
7
8
9
10
11
12
13
14
15
16
17
18
19
20
22

(h)

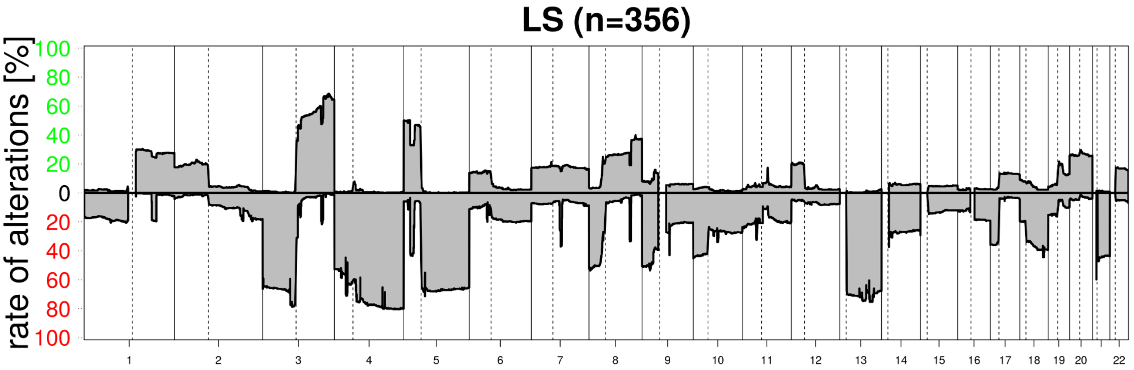
LS (n=356)
rate of alterations [%]
100
80
60
40
20
0
20
40
60
80
100
1
2
3
4
5
6
7
8
9
10
11
12
13
14
15
16
17
18
19
20
22

(i)

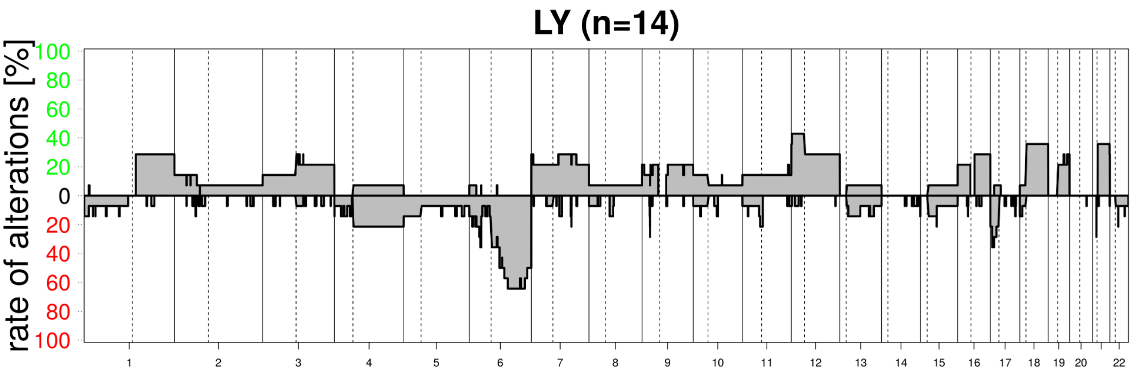

(j)

Figure S6. (a-j) Copy number summary plots created with the CNsummary package provided by Damien Stichel (https://github.com/dstichel/CNsummaryplots), which is based on a modified version of conumee (https://github.com/dstichel/conumee). Specimen types: FA=fibroadenoma, PL= benign phyllodes tumor, PM = malignant phyllodes tumor, PB = borderline phyllodes tumor, BC_Her2_norm = breast cancer without Her2 amplification, BC_Her2_ampl = breast cancer with Her-2 amplification, CB = control breast tissue, LA = lung adenocarcinoma, LS = lung squamous carcinoma, LY = diffuse large B cell lymphoma. Note that only a subset of files could be analysed by this tool (case counts indicated in plot titles). The IDs of the array data files used to generate these plots are listed in spreadsheet supplementary file S7.
