# Supplementary material for: An Integrated Epigenomic and Genomic View on Phyllodes and Phyllodes-like Breast Tumors
Source: Cancers (Basel). 2022 Jan 28;14(3):667. doi: 10.3390/cancers14030667 (PMC8833410; doi:10.3390/cancers14030667)

203293640041_R07C01

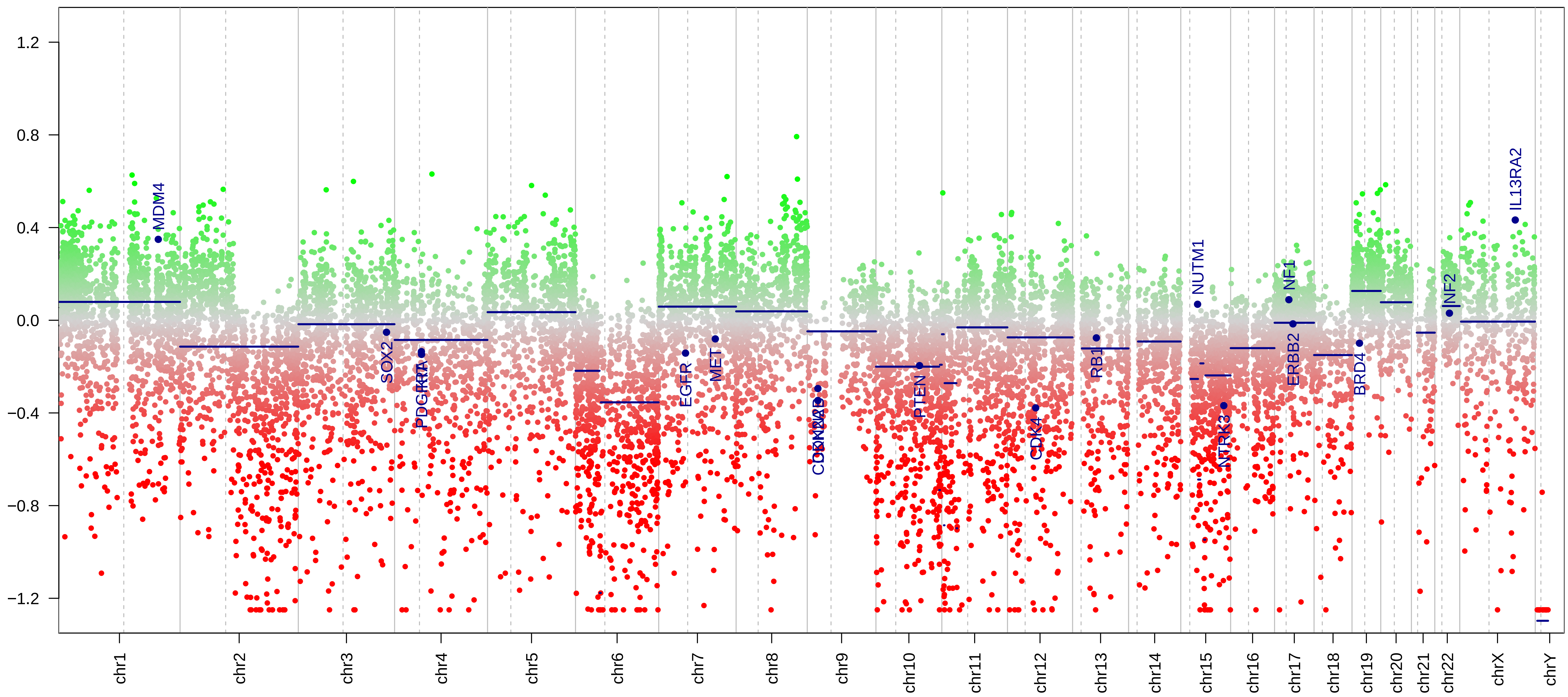

Supplement: Supplementary file 1 [file cancers-14-00667-s001.zip › S5 Nanopore_PT.zip!/S5 Nanopore_PT/203293640041_R07C01_CNV_IFPBasel_annotations.pdf]
