# Supplementary material for: An Integrated Epigenomic and Genomic View on Phyllodes and Phyllodes-like Breast Tumors
Source: Cancers (Basel). 2022 Jan 28;14(3):667. doi: 10.3390/cancers14030667 (PMC8833410; doi:10.3390/cancers14030667)

NanoDiP Report for Sample B1992_24268_20211126_BC12

Generated on	:	meqneuropat17 / 20211126_143419
Detected barcode	:	barcode12
Valid reads	:	142994
450K overlap CpG count	:	4488
Reference data	:	AllIDATv2_20210804

Methylation UMAP plot

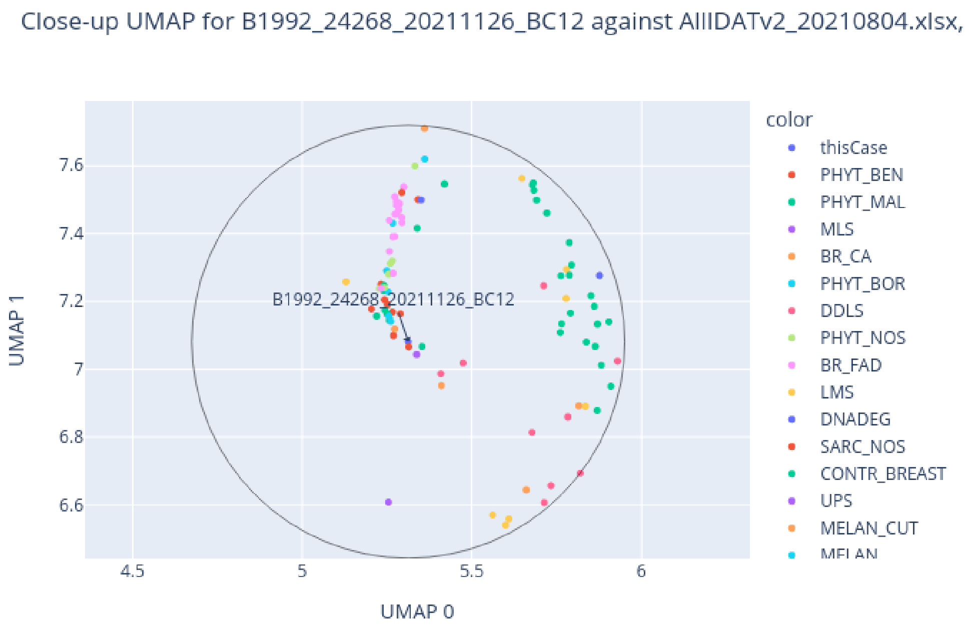

Copy number plot

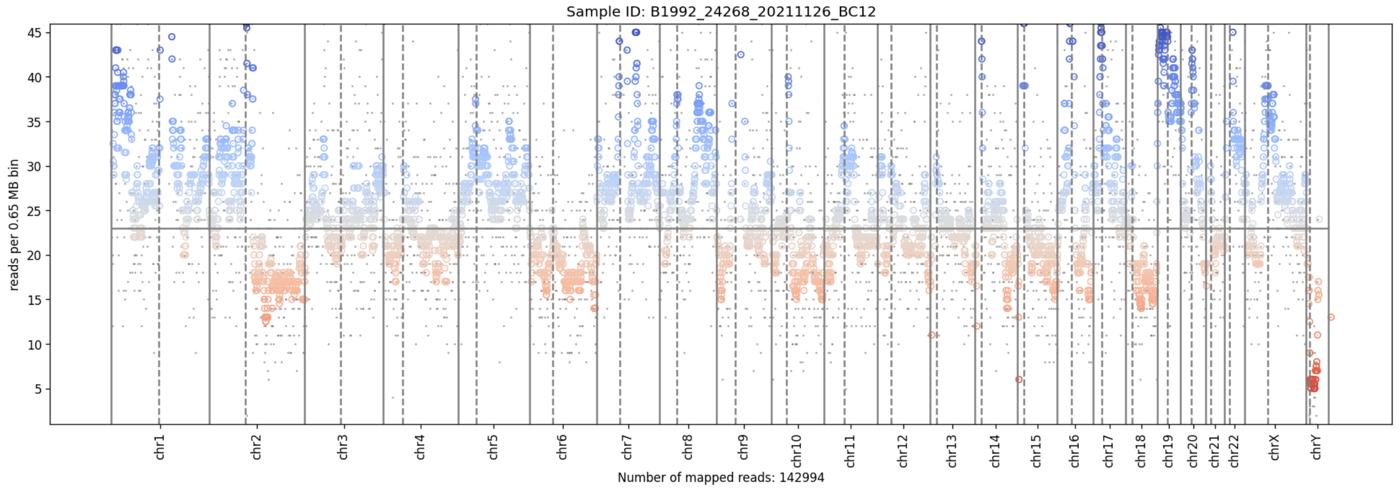

Supplement: Supplementary file 1 [file cancers-14-00667-s001.zip › S5 Nanopore_PT.zip!/S5 Nanopore_PT/B1992_24268_20211126_BC12_AllIDATv2_20210804_NanoDiP_report.pdf]
